# How general practitioners perceive and assess self-care in patients with multiple chronic conditions: a qualitative study

**DOI:** 10.1186/s12875-017-0679-0

**Published:** 2017-12-22

**Authors:** Mads Aage Toft Kristensen, Bibi Hølge-Hazelton, Frans Boch Waldorff, Ann Dorrit Guassora

**Affiliations:** 1 The Research Unit for General Practice and Section of General Practice, Department of Public Health, University of Copenhagen, Øster Farimagsgade 5, P.O. box 2099, DK-1014 Copenhagen K, Denmark; 2 Zealand University Hospital, Munkesøvej 20, DK-4000 Roskilde, Denmark; 3 Department of Regional Health Research, University of Southern Denmark, Winsløwparken 19, 3, DK-5000 Odense C, Denmark; 4 Research Unit of General Practice, Institute of Public Health, University of Southern Denmark, J.B. Winsløws Vej 9, DK-5000 Odense C, Denmark

**Keywords:** Multimorbidity, Chronic disease, Diabetes, General practice, Self-care, Continuity of patient care

## Abstract

**Background:**

It is not known how general practitioners (GPs) perceive the concept of self-care and how they assess self-care ability in patients with multiple chronic conditions. As a part of the strategy to improve the care of people living with chronic conditions, disease management programs in Denmark require GPs and other health care workers to assess and support patients’ self-care ability. The aim of the present study was to explore GPs’ perceptions and assessment of self-care ability in patients with multiple chronic conditions who have difficulty following a given treatment.

**Methods:**

A qualitative study conducted through in-depth, semi-structured interviews with a purposive sample of 12 GPs in rural areas of Denmark with economically disadvantaged populations. The interviews involved 36 complex patient cases selected by the GPs themselves. Our analysis followed the principles of systematic text condensation.

**Results:**

Most GPs in our study had a health-related perception of self-care, but some had a broader perception encompassing the situational context of the patient’s life. The GPs’ assessments of patients’ self-care ability were based on information from the ongoing and often long-term relationships with the patients. GPs identified four major factors that influenced patients’ self-care ability, which accumulated and fluctuated over time: multimorbidity, cognitive resources, material resources, and the patients’ social contexts.

**Conclusions:**

The GPs in this study had dual perceptions of self-care, related to both the chronic health conditions and to the broader situational contexts of their patients’ lives. GPs’ assessments of self-care ability depended largely on their experiences from the doctor-patient relationship, and they emphasized that the factors affecting self-care ability were highly dynamic over the patient’s lifetime. However, these findings might be resisted by the Danish disease management programs, which tend to have a static and more narrow, health-related view of patient self-care. The Danish programs require GPs to assess self-care ability upfront at the beginning of treatment and do not consider whether a relationship with the patient is established. If GPs’ perceptions and assessments of self-care ability are not included in chronic disease management models, there is a risk that they vill be insufficiently implemented in general practice.

## Background

Health care workers’ attention is drawn to patients’ self-care ability in the worldwide reorganization of chronic care to improve management of disease. In Denmark, disease management programs (DMPs) require general practitioners (GPs) to assess patients’ self-care ability in order to inform the referral process to specialists. In the case of type-2 diabetes (T2DM) [1], the DMP is already well established in Denmark and it suggests that patients with high self-care ability should follow treatment regimen as discussed with their GP or hospital specialist, whereas patients with low self-care ability should also be supported by community health care.

The DMPs describe self-care as the capacity to maintain health, to obtain quality of life, and to be responsible for treatment of chronic conditions [2]. Over time, the concept of self-care has evolved and now many different definitions exist [3] based on the priorities of the different health care professions and sectors [4]. A recent systematic review highlighted the need for further clarification of the concept of self-care [5]. Patients perceived self-care as everyday actions but were unfamiliar with the term [6]. Nurses had a strong tradition of both a clinical and a scientific use of self-care, while among doctors only GPs used the term in research of chronic care, but did not specify either its definition or its application in clinical practice. In this study, we used a pragmatic definition of self-care, which is: the GP’s perception of a patient’s ability to follow the recommended treatment.

Increasing patient complexity in terms of complicating multiple chronic conditions, psychosocial, and environmental factors tends to disrupt self-care [7]. Chronic conditions are more prevalent in economically disadvantaged areas [8] where psychological distress in patients is also higher [9] and may negatively affect the ability of self-care [10, 11]. Therefore, GPs working in such areas are of particular interest because they can be expected to have broad experience of patients with a combination of multiple chronic conditions and low self-care ability [12].

The Danish DMPs are inspired by the Chronic Care Model [13] and have been introduced for a number of chronic conditions with the purpose of improving disease management and reducing care utilization [2]. The success of DMPs depends largely on users’, typically GPs’, familiarity with the applied concepts and the required assessments of patients. Self-care is used as a central feature in the DMPs that is shared between health professionals and patients. However, we know little about how GPs make sense of this concept or how they assess self-care ability. The present study was designed to fill that gap in our knowledge.

## Methods

The aim was to explore GPs’ perceptions of self-care and, in particular, to explore how GPs assess self-care ability in patients with multiple chronic conditions and difficulty following a given treatment.

In Denmark, most people are registered with a GP for primary health care, which is free at the point of use. Limited co-payment is required for medications and some additional services such as visiting a physiotherapist or a psychologist. Most patients with common chronic conditions are treated in general practice, and patients need referral from GPs to consult specialists [14].

The GPs in our study were recruited from two rural municipalities in south-eastern Denmark with lower socio-economic status and a high prevalence of chronic conditions due to ageing populations. The sampling of GPs was a two-step process: first, a presentation by researcher, MATK, at local meetings with 20 GPs resulted in four GPs volunteering to take part in the study. Subsequently, and in order to provide maximal variation within the total sample, an additional 10 GPs were purposively selected based on their age, gender, practice size, and location. Of these, eight agreed to participate, on receipt of an invitation to take part in the study. Table 1 shows the characteristics of all 12 participants.

**Table 1:** personal and demographic details of the GPs who participated in the study, *n* = 12

Age, years. Median (range)	56	(37-69)
Gender, number		
Male	6	
Female	6	
Seniority, years. Median (range)	16	(1-41)
Practice size		
1 GP	6	
2-3 GPs	5	
> 3 GPs	1	
Practice location		
Semirural, <5000 inhabitants	3	
Urban, >5000 inhabitants	9	
Distance to Hospital		
< 30 min’ drive. N (range, minutes)	5	(2-27)
> 30 min’ drive. N (range, minutes)	7	(35-51)

In 2015, MATK, who is also a GP, conducted individual, semi-structured interviews with participating GPs at their practices. Each interview lasted for between 55 and 75 min. An interview guide provided a flexible framework for questioning and included GPs’ perceptions of self-care and experiences with the assessment of self-care ability. To ensure practical relevance and freedom to formulate thoughts and ideas from everyday examples and situations, the interviews began with a discussion about three anonymised case patients, whose files the GPs had identified in advance (36 case patient files in total). The selection criteria for the case patients were: 1) diagnosis of T2DM, 2) diagnosis of one or more other chronic conditions, and 3) experiencing difficulty in following recommended treatment. The selection of T2DM as a criterion reflects its prevalence as a common chronic condition in Denmark that is highly relevant for clinicians, particularly GPs. In Denmark, one of the first DMPs to be implemented was the program for T2DM and most general practices have systematized diabetes care in place. The case patients had common chronic conditions in addition to T2DM, and more than half of them had mental disorders or addiction problems (Table 2). The interviews were audiotaped and transcribed verbatim.

**Table 2:** profile of the patient cases that informed discussion in the GP interviews, *n* = 36

Age, years		
Mean	62.5	
Range	37-81	
Gender, number (percentage)		
Male	21	(58%)
Female	15	(42%)
Chronic Conditions, number (percentage)		
Diabetes	36	(100%)
Heart disease	18	(50%)
Mental disorder	16	(44%)
Obesity	14	(39%)
Addiction	9	(25%)
Musculoskeletal disorders	8	(22%)
Respiratory disease	4	(11%)

We used systematic text condensation in the analysis of data. This method represents a pragmatic approach inspired by phenomenological ideas, and it is suited to a cross-case analysis of a phenomenon to develop new descriptions and concepts. The procedure has four steps: 1) total impression - from chaos to themes; 2) identifying and sorting meaning units - from themes to codes; 3) condensation - from code to meaning; 4) synthesising - from condensation to descriptions and concepts [15]. The research team included three medical doctors, with and without GP training, and one nurse, all of whom identified the themes. Analysis was primarily performed by researcher MATK together with researchers ADG and BBH who mentored the process and control coded ten and three interviews respectively. Open coding by hand was used by MATK to analyze the transcripts and through comparison of these codes, the coding framework was negotiated. Meaning units were organised in documents by code groups and condensations of code groups were written. Table 3 provides an example of the analysis.

**Table 3:** Process for analyzing our data using systematic text condensation

Theme	Meaning units (relevant quotes)	Condensation	Synthesis
Understanding the concept of self-care	*Self-care is to take care of yourself in relation to your disease, but it depends on your prerequisites, which is a part of self-care; if the prerequisites are bad, then the ability of self-care is bad as well (GP 1).*	Self-care is not only related to disease.	Self-care includes all aspects of life. Health-related self-care cannot be isolated for assessment.
	*Self-care is to appreciate yourself so much that you think it is okay to spend time and effort on taking care of your health…* *I think it reflects when you are just on the ropes and you actually do not count yourself for anything. If you cannot see a way out of the problems and feel not more worth than being walked over, then why should you take your insulin? (GP 3).*	To have a positive approach to dealing with the problems you meet	
	*Regarding self-care, I thought not just following the treatment but also doing something actively to have a good life…* *It is about your ability to set the framework for your life, where you are doing fine. It is not necessarily identical to what we (GPs) think it takes to reach our goals for different chronic conditions. If you have a good life and you experience a good quality of life, I think you have more energy and are more minded to hold on to the good life by taking care of yourself (GP 8).*	To be active in creating a good life and thereby have interest in holding on to the good life, despite of health challenges	
	*If you are unemployed, have personal financial problems, and hardly can make a living, you simply have no energy to care for yourself. You must be in control with the basic stuff before you can start changing your lifestyle (GP 11).*	The basic stuff goes before lifestyle changes	
	*What is their ability to make a move? Some of them do not have it… No matter what we come up with, they have no energy to do something about it. Maybe they cannot see it or maybe they do not have the resources; then it is just easier to take the usual course because they have no energy to change anything. They have a lack of resources, in terms of economy, socially and mentally, to be able to change it (GP 12).*	Improving self-care depends on the resources to make a move	

Self-care entails a diverse understanding and a broad consensus at the same time, in that we all understand what is meant by self-care, and yet the concept is highly dependent on patient circumstances and the level of health care. Therefore, self-care fits the concept of a boundary object, which forms the theoretical background of the present study. Boundary objects are defined as objects, which are both plastic enough to adapt to the local needs and constraints of the parties employing them, yet robust enough to maintain a common identity across sites, in our case, across sector boundaries within the Danish health care system [16]. This means that the broad use of the term self-care is likely to cover several different local understandings among the actors involved in DMPs, including understandings in general practice, as well as aiming toward some workable objectives in the management of chronic health conditions.. Other theories were brought into the analysis, for example Orem’s theories of self-care and health literacy.

## Results

Our analysis suggested that most of the GPs we interviewed perceived self-care as being solely related to health, but some GPs found that it related to all other aspects of the patient’s life. The GPs’ assessments of patients’ self-care ability were based on knowledge obtained through the ongoing doctor-patient relationship. In their assessments, GPs identified four major factors: multimorbidity, cognitive resources, material resources, and social context. Each one of these factors contributed to limiting patients’ capacity to take care of themselves, either permanently or for a period of time. Some patients struggled with challenges from two, three, or even all four of them.

### Self-care – limited to health or including all aspects of the patient’s life

Most of the GPs perceived self-care as the patients’ ability to take care of their health and these GPs identified three successive prerequisites of patients’ self-care ability: 1) recognizing a health problem and viewing it in the context of the total life situation; 2) giving priority to the health problem and willingness to spend time and effort on adapting life to the health problem; 3) carrying out and adhering to the adaptations made to address the health problem.

*Self-care is the patient’s ability to look after the disease. A good ability of self-care is to be able to understand what the disease is about and how to relate to it. But especially to be able to carry through changes and do what is necessary. Having diabetes, it’s about exercising and eating healthy and managing the medical check-ups and the medications (GP 5).*

A substantial minority of GPs also defined self-care as belonging to more domains of a patient’s life; health related self-care is only part of the ability to take care of oneself. Other aspects for patients were to recognize, prioritize, and take proper measure of their total life situation in order to make disease and its treatment fit into daily life, including, for example, social networks and work life. Self-care was also seen as a proactive approach to solving the challenges in life, where a positive outlook could be very helpful. One GP connected self-care to self-appraisal: does the patient appreciate himself or herself enough to make the effort to care for his or her health?

*To be able to take care of some of the things, that we (as GPs) want people to take care of, it is very important that you think you have a good life and experience a good quality of life. Then, I think, that you feel more in control and want to hold on to the good life by taking care of yourself (GP 8).*

Some of the GPs could not describe the meaning of self-care, but one GP stated that he was confident that an unspoken awareness of self-care played a role when he considered the patient’s ability to follow a complicated medical treatment like injection of insulin.

### GPs’ evaluation of self-care ability and the ongoing doctor-patient relationship

The GPs got most of their knowledge of a patient’s self-care ability from their ongoing relationship with the patient. They knew patterns from earlier disease trajectories, current adherence to medications or appointments, and the patient’s reaction to advice on lifestyle changes, such as weight loss or cessation of smoking. In smaller communities, some GPs had knowledge of the patient’s everyday life in the community.

*When you have known people for so many years then you really do not need to ask very much about self-care, because you know their work situation, who they are married to, their children and all these things. I really do not sit writing if they manage one or another thing (GP 4).*

The GPs were reluctant to assess self-care ability at the first meeting with patients they did not already know and waited for later meetings to get a better assessment from the patients’ questions and their feedback on changes in lifestyle. However, even with new patients, GPs observed signs of self-care ability.

*I attach importance to how they move, their size, and how worn-out they look. And how they express themselves. It is just like this; if you are not too smart, then it can be difficult, and you might get a sense of that from the conversation (GP 2).*

### Major factors influencing the ability of self-care

According to the GPs, the ability of self-care could vary over time due to changes in both health and life circumstances. A number of factors affected self-care – in both negative and positive ways over time, as illustrated below.

*You can say that it (the self-care ability) fluctuates: I had a patient here, who didn’t monitor her diabetes for two years. I think it was because her marriage had failed and she had gotten divorced. Then she met a new man and things changed. She started exercising and began to take her insulin again (GP 3).*

However, the most important factors influencing a patient’s self-care ability, according to the GPs in this study were: multimorbidity, cognitive resources, material resources, and social context.

#### Multimorbidity

In the GPs’ experience, one condition could act as a barrier to self-care of another disease. If a patient with diabetes had respiratory problems from a lung disease, it could be difficult to exercise as much as the GP recommended.

*She is simply very obese… and then her back is so bad, that she has to take a lot of painkillers as well, so she has such pain in her body, and it results in a total absence of exercise… I think that she hardly can walk from the waiting room to the next room with a walker. She is really in a bad position because of her weight and her pain (GP 1).*

The presence of a high number of concurrent chronic conditions could even block the attention of both the GP and the patient to some of the diseases.

*I believe this patient has seven chronic conditions, really significant diseases, and one day we realized that we had not discussed his severe COPD for four years. Because he has diabetes, atrial fibrillation, rheumatoid artritis and ... I am not able to remember all of them, but the COPD was completely forgotten (GP 3).*

Some of the GPs found that patients with limited personal resources were less inclined to overcome the barriers of disease. One GP described an unemployed patient with diabetes who seemed to focus on the pain from arthrosis in her knees instead of finding alternative ways to exercise. Another GP also experienced patients who were not able to by-pass physical limitations in order to exercise more.

*Yes I do (talk to the patient about his lack of exercise), and then of course he says like the previous patient: ‘I just can’t walk, because I have pain in my back’. He likes to go fishing and he thinks that he gets much exercise from that, although his boat is small, so that’s not much. Of course, he lives his life as he thinks it should be lived. I have talked much to him about walking or biking or swimming, but no (GP 2).*

Patients with a combination of somatic and severe psychiatric disorders or abuse of alcohol had particular difficulties. For example, one GP had a patient with diabetes and an anxiety disorder who could not participate in the lay-led diabetes program. In the GPs’ opinion, somatic conditions could not be treated unless the alcohol abuse or psychiatric disorder were well under control. Self-care related to somatic treatment could fluctuate depending on how the patient was responding to treatment for psychiatric disorders and substance abuse.

*In his case, as soon as his psychiatric disorder is well treated and he does not drink alcohol, he actually has a decent ability of self-care. He is average clever; he has just always had a psychiatric disorder. Thus, he understands that he has to take care of his diabetes … (GP 6).*

#### Cognitive resources

Most of the GPs had experience of patients with limited educational attainment through either lowered intellectual capacity or from mental stress or dementia. The GPs found that self-care ability in these patients was lower because they often had additional social problems, had unhealthy habits, or were unable to change their lifestyles.

A limited knowledge and understanding of the nature of the disease and the body could lead to misunderstandings between the patient and the GP about the management of chronic conditions. The GP repeated the same advice to patients who easily lost the overview and focused on less relevant aspects of self-care. Therefore, the GP’s ambitions for treatment could be lowered. The GPs perceived that some of these patients who had no disease symptoms, for example from early stage diabetes, did not take the disease seriously or even neglected it.

*It’s not that easy to… explain things to her… If we discuss a healthy diet: do you eat any vegetables? Yes, I eat one tomato a day. Therefore, she is really in a completely different space than she should be in terms of just about everything (GP 1).*

#### Material resources

Some patients’ residential conditions were so miserable that they directly affected disease. In the case illustrated below, this frustrated the GP:

*When his damned asthma is not bad, when it is not season for his asthma, then the diabetes and everything works fine. His current problem is that (his home has) a very bad indoor climate that worsens his asthma. That is what turns him over again and again… he might be admitted to the hospital two times in three months (GP 4).*

Many patients with low income could not afford the expenses of transportation, medication, or additional treatments with a limited co-payment. In the setting of our study, many of the patients lived in rural areas with long distances to travel to the GP or to sport facilities and with no opportunity for public transport.

*The finances play a very great role… Because if you have three chronic conditions, despite the maximal governmental grants, then there is a co-payment of 50 Euros per month. It can be too much for them, and then they will not buy the medications, they simply cannot afford the medications… I have certainly some examples of that (GP 12).*

#### Social context

The GPs found that social context could enhance or limit the ability of self-care. Dietary habits had a great influence on many diseases but were difficult to change if the spouse, or person responsible for cooking in the household, was not supportive.

*A man with an alcohol use disorder, who lives alone, is often a bit more difficult to reach than others are. But then again, men with wives, who are incapable of adjusting their eating habits, these men can almost be even more difficult to treat. Social circumstance plays a major role in self-care (GP 2).*

Problems in the patient’s close relationships, like divorce or serious disease in a partner or child, could also disturb the ability of self-care fundamentally for a period of time, or permanently.

*I told her, that her numbers (blood glucose) had worsened. Then she said by herself: ‘Yes, but it is about my (child), who is ill and has just been admitted to the hospital’. Then I said: ‘but yes, I understand’. That is just the advantage of knowing the family... I know their life stories, so I can easily see the whole picture (GP 12).*

Other problems in the patients’ social context might drain the resources needed to change lifestyle. Sometimes GPs had to accept that due to the patients’ social contexts, self-care ability was not sufficiently present to follow the guideline treatments for some diseases.

*If you have problems at work, you clearly have more prominent and important challenges than changing your lifestyle and exercising. Obviously, if you hardly can manage your everyday, you simply have no extra energy. You just need to be in control with the basic stuff before you are able to care for yourself in terms of life style changes (GP 11).*

## Discussion

In this study, two perceptions of self-care prevailed among the GPs: a health-related perception and a broader perception that encompassed all aspects of the patient’s life. The assessment of self-care ability was based on information from the ongoing relationship with the patient. The GPs described four major factors that influenced self-care ability that both accumulated and fluctuated over time: multimorbidity, cognitive resources, material resources, and the patient’s social context.

### Limitations

We were aware of the risk of conceptual blindness due to peer interviewing, therefore in an attempt to reduce this risk, our research team included health professionals from outside general practice in the planning and analysis of the study [17, 18]. The participating GPs knew the interviewer’s identity as both a GP and a researcher, which could account for the high participation rate. The GPs said that they felt the interviews were confidential and that they could talk more directly to a peer about complex matters of clinical practice than to an interviewer from another professional background. The GPs’ candid responses are well illustrated in this article, and they may not have been so forthcoming with an interviewer outside their peer group.

This study took place in two economically disadvantaged rural areas of Denmark, which might raise a question about the transferability of the findings to other settings. However, an English study found that GPs practicing across a wide range of socio-economic demographics had similar experiences when faced with the challenges of multimorbidity. This would suggest that pessimistic attitudes in this context are more likely to be a response to dealing with complex patients than to working with patients of lower socio-economic status [19]. Our results therefore could be applied to similar patients in areas with different socio-economic characteristics.

The use of real patient cases during the GP interviews may have introduced an element of bias, but it also enriched the interviews by allowing the GPs to articulate their thoughts and experiences with an independent peer researcher. It also reduced speculation by the GPs about the background characteristics of fictional patients. All of the selected case patients had difficulty in following the recommended treatments for one or more of their chronic conditions. Therefore, the factors we identified that affected self-care ability might be more focused on the barriers to self-care, rather than on the resources supportive of self-care. However, the GPs’ perceptions on self-care did not relate exclusively to multimorbidity and most of the findings are also applicable to patients with single chronic diseases.

### Comparison with existing literature

We have found few studies describing how GPs assess self-care ability in patients with one or multiple chronic conditions. However, our study showed that GPs used information from their ongoing relationship with their patients to make their assessments, and this finding is in line with results from other studies. Continuity in the doctor-patient relationship plays an important role in chronic care [20], especially for patients with complex chronic conditions who can be reluctant to visit their practice if they are not seeing the same doctor [19]. General practice has a tradition for developing longer doctor-patient relationships and for using a patient-centred clinical method that includes understanding the patient as a whole person [21]. The current shortage of GPs in Denmark and in other countries challenges this continuity as an increasing number of GPs are employed on a short-term basis.

The GPs’ health-related perceptions of self-care in our study are comparable to an English study where GPs viewed self-care as comprising many different health behaviours summarized into three themes: appropriate help-seeking, compliance with medication, and healthy lifestyle choices [19]. In our study, some of the GPs perceived that self-care ability involved more than the health-related aspects of the patient’s life. This openness to the full range of difficulties patients bring to their doctors, and not just their biomedical problems, is more in line with patients’ perceptions of the challenges of chronic conditions. Many patients have greater difficulty dealing with the physical and emotional symptoms of chronic disease than with the medical tasks [22].

The GPs in our study emphasized the highly dynamic nature of self-care ability in their patients with multiple chronic conditions. Longitudinal interview studies with patients support this experience [23, 24] showing that self-care ability tended to fluctuate more in patients with multimorbidity, due to, for example, exacerbation of one condition, contradictory information about conditions, or work overload caused by appointments [25]. A Scottish study of GPs’ experiences of patients with multimorbidity areas of lower socio-economic status found that self-care ability in most of the patients was frequently and recurrently disrupted by complicating medical and social problems [10].

The GPs’ in our study described health-related self-care as the ability to recognize, prioritize, and adapt to a health problem. These three steps are comparable to the definition of health literacy, which comprises the ability of the patient to process health information [26]. However, the GPs’ perception of health-related self-care also included the ability to take actions in life, which the GPs often saw as the patients’ most challenging task. The GPs in this study recognized patients’ limited educational attainment, in terms of deficient knowledge and understanding of disease, as a fundamental barrier to self-care. Tools to assess health literacy might be useful in clinical work with self-care, but they are criticized for underestimating the complexity of health literacy by focusing on the patients’ ability to read and understand written materials [27]. In a clinical setting, the GPs in our study pointed out that patients’ contextual factors in terms of psychosocial matters had a major impact on self-care ability. In many cases, accounting for patients’ health literacy alone might be insufficient to improve self-care.

### Comparison with other theoretical approaches

In our study, the GPs’ perceptions of self-care were similar but did not refer to any specific theoretical model. In the assessment of self-care ability, GPs included the integration of experiences with patients’ initial attempts to change lifestyle and patients’ understanding of instructions. Orem’s theories of self-care are some of the most cited in the literature. Orem defined self-care as the practice of activities that an individual performs on their own behalf in maintaining life, health, and well-being. Self-care relates to the needs of individual patients [28] and the ratio of the patient’s self-care demand and self-care agency indicates whether or not self-care is sufficient. This model is similar to the perceptions of the GPs in our study in terms of the fluctuating balance over time and the factors influencing self-care. Orem’s theories originate from the context of nursing and relate to people with a need for nursing care because of disease or general weakness. In general practice, most patients have limited access to nursing assistance and the GP uses the assessment of self-care ability in deciding on the appropriate treatment, which thereby affects the burden of treatment for the patient [29]. In Orem’s words, this is the therapeutic self-care demand. Sometimes a reduced capacity for self-care compromises the intended treatment, which is often the case for patients with multiple chronic conditions where the patient and the GP may decide to deviate from the treatment guidelines [30]. From a general practice perspective, Orem’s theories of self-care seem to focus on health-related self-care and omit the broader perspective of self-care that enhances prevention and promotes health and well-being [31] that some of the GPs in our study perceived as important.

### Implications

In this study, how GPs assess patients’ self-care may conflict with the directions of DMPs in several respects. First, the GPs base their assessments on their accumulated experiences with their patients over time, while the DMPs expect GPs to make an upfront assessment of self-care ability from the beginning of the treatment, regardless of the continuity in the doctor-patient relationship. Moreover, GPs emphasize the significance of a broad, whole person approach to patients and the dynamic nature of self-care ability, whereas the DMPs tend to view patients’ self-care ability in a narrow, health-related way and as a rather static characteristic. In patients with several concurrent chronic conditions, the treatment guidelines in the DMPs may accumulate, duplicate, or even conflict, which makes them complicated to use in practice, and more difficult for patients to follow. In these ways, the DMPs do not correspond to basic elements of GPs’ perceptions of self-care, which might negatively affect the GPs’ appraisal of the DMPs’ relevance. A study from England found that when the emphasis of health policies on self-care support conflicted with GPs’ experiences of self-care, the GPs were reluctant to discuss self-care with patients [32]. In this light, these conflicts raises a degree of uncertainty about GPs’ use of DMPs in Denmark. If self-care is perceived differently by the health professionals who are supposed to collaborate on patient care, then there must inevitably be concerns about the quality of the cross-sectoral care of patients with complex, chronic conditions. In Denmark, DMPs are intended to organize care for the chronically ill and to facilitate cooperation between all of the actors involved in that care, therefore a sufficiently robust translation between local understandings of the concept of self-care is needed [16]. This does not seem to be the case for DMPs in Denmark.

To further the research insights we have identified, an investigation of GPs’ use of and attitudes towards the DMPs is needed. An observational study of GPs’ consultations with patients with multiple chronic conditions would also provide more knowledge about the clinical assessment of self-care ability and how patients are involved. Finally, patients’ perspectives of self-care ability should explore the accuracy of GPs’ assessments. This is currently unknown but is surely desirable since a working consensus is needed for successful outcomes.

## Conclusions

Two perceptions of self-care prevailed among the GPs in this study: a health-related perception and a broad perception that emphasized the significance of patients’ situational contexts for the ability to self-care. GPs’ assessments were based on information from the ongoing relationship with the patient, and four major factors were found to influence perceptions of self-care ability: multimorbidity, cognitive resources, material resources, and the social context, all of which accumulated and fluctuated over time.

These findings are important since little is known about how GPs perceive and assess self-care. Worldwide, chronic care is being reorganized around disease management models that require health care workers to assess and support patients’ self-care ability. This study suggests that the DMPs in Denmark may conflict with GPs’ perception and assessment of self-care in patients with multiple chronic conditions in several key respects: continuity in the doctor-patient relationship, the dynamic nature of the patients’ situational context over time, and dramatic fluctuations in self-care ability that may occur when new chronic conditions are diagnosed or existing conditions are exacerbated. The current DMPs in Denmark do not seem to reflect GPs’ perceptions of self-care. This might negatively affect GPs’ sense of relevance and the total effect of the DMPs.

The potential benefits from chronic disease management models are substantial for patients and health care systems. However, if GPs’ perceptions of self-care and the assessment process are not included in these models, there is a risk of ineffective implementation in general practice, where the majority of patients with chronic conditions receive care. The quality of cross-sectorial care of patients with chronic conditions might also be reduced if differences in the perceptions of self-care between collaborating health professionals are not taken into account.

## Abbreviations

DMP: Disease management program
GP: General practitioner
T2DM: Type 2 diabetes mellitus
